# Effect of different vegetation restoration patterns on community structure and co-occurrence networks of soil fungi in the karst region

**DOI:** 10.3389/fpls.2024.1440951

**Published:** 2024-09-04

**Authors:** Xiaoxiao Zou, Kai Yao, Zhaoxia Zeng, Fuping Zeng, Lihong Lu, Hao Zhang

**Affiliations:** ^1^ Key Laboratory of Agro-Ecological Processes in Subtropical Region, Institute of Subtropical Agriculture, Chinese Academy of Sciences, Changsha, China; ^2^ Karst Dynamics Laboratory, Ministry of Natural Resources, Institute of Karst Geology, Chinese Academy of Geological Sciences, Guilin, China; ^3^ School of Life Science, Guizhou Normal University, Guiyang, China; ^4^ Huanjiang Observation and Research Station for Karst Ecosystem, Chinese Academy of Sciences, Huanjiang, China

**Keywords:** karst ecosystem, fungal community, grain for green project, co-occurrence network, assemble mechanism

## Abstract

**Introduction:**

The Grain for Green Project (GGP) by the Chinese government was an important vegetation restoration project in ecologically fragile and severely degraded karst regions. Soil fungi play a facilitating role in the cycling of nutrients both above and below the ground, which is crucial for maintaining ecosystem function and stability. In karst regions, their role is particularly critical due to the unique geological and soil characteristics, as they mitigate soil erosion, enhance soil fertility, and promote vegetation growth. However, little is known about how the implementation of this project shifts the co-occurrence network topological features and assembly processes of karst soil fungi, which limits our further understanding of karst vegetation restoration.

**Methods:**

By using MiSeq high-throughput sequencing combined with null model analysis technology, we detected community diversity, composition, co-occurrence networks, and assembly mechanisms of soil fungi under three GGP patterns (crop, grassland, and plantation) in the southwestern karst region.

**Results:**

Ascomycota and Basidiomycota were the main fungal phyla in all the karst soils. Returning crop to plantation and grassland had no significant effect on α diversity of soil fungi (*P* > 0.05), but did significantly affect the *β* diversity (*P* = 0.001). Soil moisture and total nitrogen (TN) were the main factors affecting the community structure of soil fungi. Compared with crop, soil fungi networks in grassland and plantation exhibited a higher nodes, edges, degree, and relatively larger network size, indicating that vegetation restoration enhanced fungal interactions. The soil fungi networks in grassland and plantation were more connected than those in crop, implying that the interaction between species was further strengthened after returning the crop to plantation and grassland. In addition, null-model analysis showed that the assembly process of soil fungal communities from crop to grassland and plantation shifted from an undominant process to dispersal limitation.

**Discussion:**

These data indicated that GGP in karst region changed the composition and assembly mechanisms of the soil fungal community and enhanced the interaction between fungal species, which can contribute to a better understanding of the fungal mechanisms involved in the restoration of degraded karst soils through vegetation recovery.

## 1 Introduction

Karst regions cover 12% of the total global land area, as one of the most ecologically fragile zones in the world, the ecological and environmental issues have attracted increasing international geoscience research (Parise et al., 2009; Wang et al., 2019b; Chen et al., 2020). In the contiguous and extensive karst regions of southwestern China, including Yunnan, Guizhou, and Guangxi provinces, the area affected by rocky desertification has reached 113,500 km^2^, accounting for 53.4% of the total rocky desertification in the country (Jiang et al., 2014; Hu et al., 2020; Zhang et al., 2022b). Influenced by both natural factors (easy solubility, shallow soil, discontinuous soil cover, and slow soil formation, etc.) and human factors (excessive development and irrational farming practices, etc.), severe vegetation degradation was widespread in most areas, posing a direct threat to the ecological security of the upstream Yangtze and Pearl River basins (Cheng et al., 2017; Yan et al., 2018). To effectively reduce rocky desertification, the Grain for Green Project (GGP) launched by the China government to return crops to forests and grasslands in karst regions from 1999 (Chen et al., 2020; Hu et al., 2020; Zhang et al., 2022a). As of now, the reduction of cultivated areas on steep slopes in the southwestern karst region has resulted in newly added areas of plantation and artificial pasture, exceeding 417,400 hm^2^ and 77,500 hm^2^, respectively (Luo et al., 2021). Despite the implementation of these ecological projects, they have, to some extent, curbed the degradation of the ecosystem (Wang et al., 2019b). However, vegetation restoration by GGP in karst region is still difficult due to the dual structure of the surface and subsurface (Breitenbach, 2008), shallow and discontinuous soil layers (Lu et al., 2022), small total amount of soil, low water storage capacity (Yan et al., 2018), and severe seasonal drought (Chen et al., 2020). Thus, vegetation recovery is mainly characterized by dwarf forests and shrub thickets, and it is highly susceptible to widespread vegetation death in extremely arid conditions (Parise et al., 2009; Zhao et al., 2017). One of the main reasons for this may be that the restoration of the ecological function of karst soil lags behind the restoration of vegetation, which makes it difficult for the restored soil to meet the growth demands of the restored vegetation (Hertog and Turnhout, 2018). Therefore, accelerating self-recovery of the soil’s ecological functions has become key to vegetation restoration in karst regions.

As the engine of biogeochemical cycles, soil microbes play an irreplaceable role in driving the soil exchange with other spherical materials, enhancing soil fertility and maintaining the stability of ecosystem functions (Hartmann et al., 2015; Shi et al., 2016). For example, soil fungal communities can degrade and transform complex organic matter into simpler compounds that can be absorbed and utilized by other organisms, participating in the processes of humification and mineralization in the soil, and playing a very important role in the formation of humus and soil aggregate structure (Chen et al., 2020). Additionally, the variations in composition, structure, and spatial distribution patterns of microbial communities can reflect changes in their habitats. Thus, investigating the represent in fungal community composition and diversity patterns can characterize the degree of recovery of karst degraded soil (Zak et al., 2003; Shi et al., 2016). Recent studies have delved deeply into the impact of karst desertification on the structure and composition of soil fungal communities, and indicated that the concentrations of exchangeable calcium and magnesium ions, total potassium, and soil temperature are important environmental factors that regulate the structure and distribution of fungal communities in karst forest soils (Chen et al., 2020; Hu et al., 2020; Lu et al., 2022). However, the research on the spatial distribution patterns (assembly mechanisms) of these communities is not comprehensive enough. Stochastic and deterministic processes are two important mechanisms in soil microbial community assembly (Jiao and Lu, 2020). Deterministic processes are dominated by abiotic factors (such as pH and temperature) and biotic factors (interspecies competition, predation, and other relationships) (Tripathi et al., 2018; Zhang et al., 2019; Jiao and Lu, 2020), and are mainly attributed to microorganisms in different habitats and adaptability. Nevertheless, stochastic processes focus on the role of diffusion and ecological drift (Zhang et al., 2022d). A null model based on the entire community has been developed to quantify the assembly processes of various communities (Stegen et al., 2013). This model not only quantitatively elucidates the ecological mechanisms of soil microbes’ co-emergence under different vegetation restoration patterns, but also aids in assessing the assembly and succession processes within soil microbiology (Barberan et al., 2014). However, the interaction between soil and vegetation is extremely complex in karst ecological restoration process, which was not only limited by the highly heterogeneous karst niche but also influenced by vegetation types, and jointly affect the interaction of soil-vegetation systems (Mori et al., 2018). Hence, a comprehensive understanding of interactions between soil and vegetation is of great significance crucial for vegetation restoration in karst areas.

Network analysis has become an important analytical method within the field of microbial ecology, because it can identify key species in community networks by determining the locations of highly connected species within the microbial network (Ma et al., 2021). Furthermore, network analysis can be used to determine the complexity and stability of the microbial community, offering new insights into the dynamics of microbial ecosystems (Zhou et al., 2020; Qiu et al., 2021). Several studies have shown that highly complex ecological networks have greater network stability and a strong ability to cope with environmental stress (Yuan et al., 2021; Xu et al., 2022; Li et al., 2023). Additionally, the functionality of desert soil was mainly regulated by microbial diversity, especially the diversity of fungal communities (Xu et al., 2022). Based on this, we hypothesized that the complexity of fungal communities in karst soil was relatively lower in more disturbed crop, while it is relatively higher in less disturbed grasslands and plantations. Our research group has shown that different restoration patterns influence the diversity of karst soil bacteria and that the assembly process is mainly stochastic (Zou et al., 2023). Unlike bacteria, fungi, serving as essential connectors between vegetation roots and soil moisture and nutrients, are speculated to exhibit responses to restoration that differ from those of bacteria (Wang et al., 2019a). However, it remains unclear how different restoration patterns alter the diversity of fungal communities and the interactions between fungal species in karst soils. In the present research, we examined the diversity, co-occurrence networks, and assembly mechanisms of fungal communities using MiSeq high-throughput sequencing combined with null model analysis technology. The following two questions were explored: (1) How do the diversity and co-occurrence networks of soil fungi change after the conversion of crop to plantation and grassland? (2) Which stochastic and deterministic processes dominate in the community assembly of karst soil fungi? This study aimed to provide a scientific basis for further understanding the ecological adaptation strategies by analyzing the diversity and co-occurrence networks of soil fungal communities in the soils of crop, grassland, and plantation of karst regions.

## 2 Materials and methods

### 2.1 Study site

**Figure 1:**
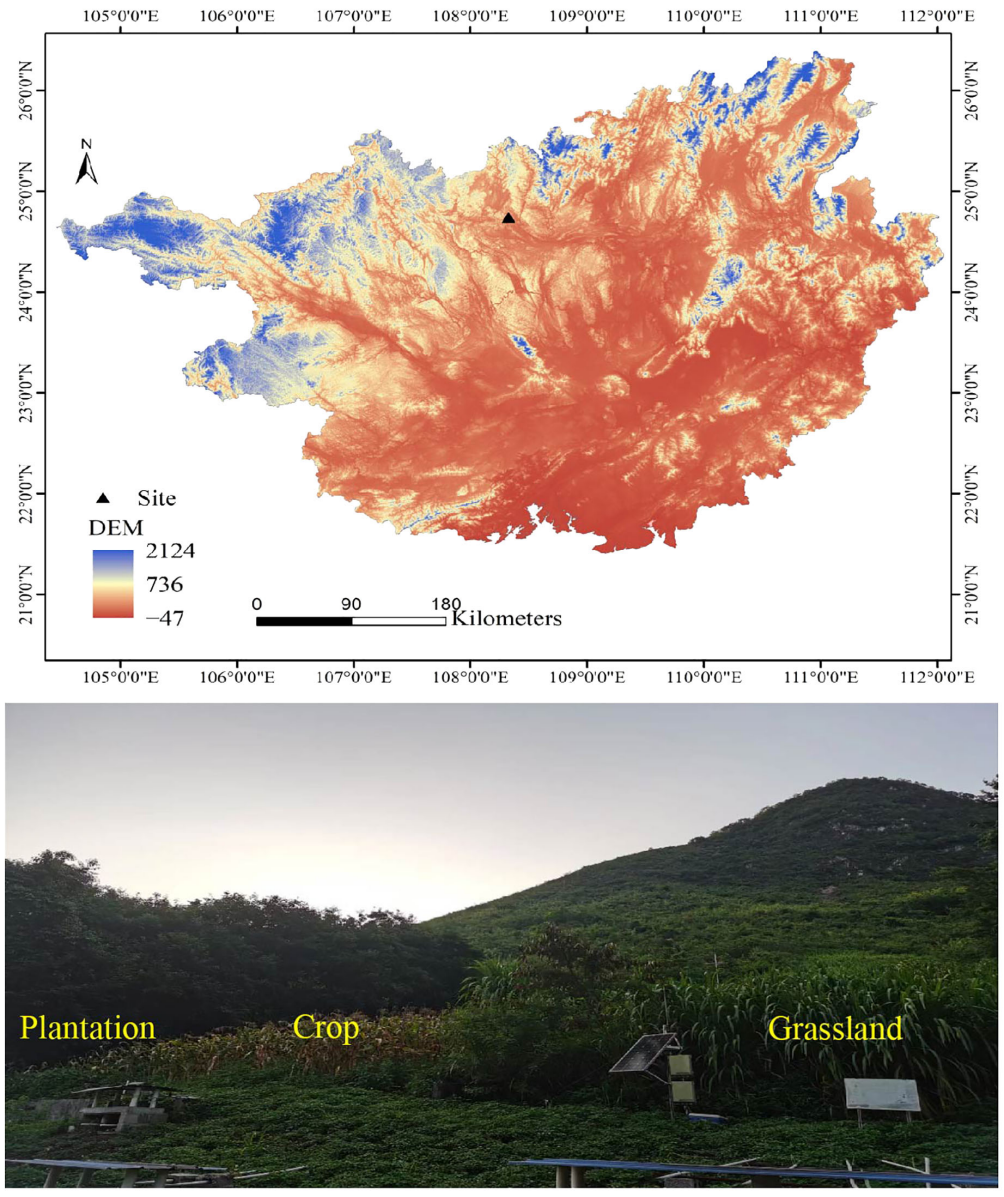
The geographic location and photos of the study area in Guangxi Province, China. DEM, digital elevation model.

The research site is located at Huanjiang Karst Ecosystem Observation and Research Station, Chinese Academy of Sciences, Guangxi Zhuang Autonomous Region, China (108°18′ E–108°20′ E, 24°43′ N–24°45′ N, average altitude 228.5–337.8 m). This area is north of the Tropic of Cancer and southeast of the Yunnan-Guizhou Plateau, which is a typical karst peak cluster depression geomorphic area. The climate is a subtropical monsoon climate, which has an annual average temperature of 19.9°C, an annual sunshine duration of 4,422 hours, annual solar radiation of 98.89 kJ·cm^-2^, a frost period lasting for 75 days, and mean annual rainfall ranging between 1,400 mm and 1,500 mm. The rainy season in this region occurs from April to September each year, accounting for 70% of the annual rainfall, and the annual evaporation is 1,380 mm. Seventy percent of the sloping land in this area had a slope greater than 25°, with a bedrock exposure rate of less than 10%. The area faces southeast and has an elevation of approximately 300 meters. The soil depth in the middle and upper slopes was 10-50 cm, and the soil depth at the bottom was 20–160 cm. Dark or brown calcareous soil developed from dolomite, with mixed gravel (>2 mm) content reaching 10–40% (Lu et al., 2022). In 2004, our research group established different experimental plots to return crop to plantation and grassland, and the size of each plot in the different restoration patterns was 70 × 20 m. All sample plots have a slope of around 25 degrees, facing southeast, at an elevation of approximately 300 meters. In the century before establishing the sample plots, the land was mainly used for cultivating crops (soybean-corn rotation), employing the same agricultural management practices (**Figure 1**) (Xiao et al., 2021).

### 2.2 Soil sampling and determination of soil nutrient

In July 2022, different vegetation restoration plots were selected from the long-term experimental plots, which included *Zea mays* L. (crop), *Pennisetum purpureum Schumach* × *Pennisetum alopecuroides* (L.) (Guimu-1 elephant grass, grassland) and *Swida wilsoniana* (plantation). Two seasons of corn were sown in February and August of each year. The inorganic fertilizers applied were urea, calcium magnesium phosphate, and potassium chloride, with the total application rates for N, P_2_O_5_, and K_2_O being 160 kg·hm^-2^, 90 kg·hm^-2^, and 90 kg·hm^-2^, respectively. the total amounts of nitrogen (N), phosphorus (P_2_O_5_), and potassium (K_2_O) were 160 kg·hm^-2^, 90 kg·hm^-2^ and 90 kg·hm^-2^, respectively. After each harvest, corn stalks were cut and taken out of the field to be crushed and used as feed. There was only a small amount of litter remaining on the ground surface. The grass is a perennial plant that does not produce seeds, and it reproduces through buds, requiring 30,000 to 45,000 buds hm^-2^. Guimu-1 is suitable for warm and humid climate, with fast growth and high yield, has significant economic value. It has been widely used in reforestation and grassland restoration projects in the hilly and mountainous regions of southern China (Hu et al., 2020). The grassland was tilled and replanted at four-year intervals, and 300 kg·hm^-2^ of N: P_2_O_5_: K_2_O = 15:15:15 compound fertilizer was applied every year after the grassland returned to green. Grass above 10 cm was harvested and fed directly to livestock, or crushed and fermented and used as silage. There is only a small amount of litter remaining on the ground surface. *Swida wilsoniana* is a resilient oil-bearing woody plant widely distributed in the southern karst regions. It has been extensively used in afforestation on rocky mountains. The plantations were closed and managed after planting. The understory shrub layer includes species such as *Alchornea trewioides*, *Rhus chinensis*, *Mallotus barbatus*, *Pyracantha fortuneana*, *Zanthoxylum armatum*, and others. The understory herbaceous layer consists of plants like *Nephrolepis cordifolia*, *Microstegium fasciculatum*, *Selaginella uncinate*, *Lygodium japonicum*, and others. The tree and shrub layers of the plantation are both composed of evergreen plants, and the understory vegetation is sparse, resulting in only a small amount of litter on the forest floor.

Three 20 m × 20 m standard squares were arranged at the top of each sample plot, and each square was divided into four sampling points in the range of 10 m × 10 m. A total of 36 (3 types × 12 replicates) soil samples were obtained at each sampling point following the 5-point plum blossom mixture sampling method for all three vegetation restoration areas. Soil samples from the 0–20 cm layer were collected using an earth drill with a diameter of 5 cm. Following the removal of visible impurities like plant roots, litter, and gravel, the samples were stored in a low-temperature foam box. Subsequently, the soil samples underwent a 2 mm screening process in the laboratory. One set was preserved in an ultralow-temperature refrigerator (−80°C) for DNA extraction, while the other set was allowed to air-dry naturally indoors for soil chemistry analysis. Soil pH, soil moisture, total nitrogen (TN), available nitrogen (AN), total phosphorus (TP), available phosphorus (AP), and soil organic matter (SOM) of the soil samples were determined as described by Bao (Bao, 2000).

### 2.3 Extraction of soil DNA and analysis of high-throughput sequencing

For each soil sample, fresh soil (0.5 g) was collected and DNA was extracted according to the Fast DNA^®^SPIN Kit for Soil, MP (Qbiogene Inc., USA) procedure. The quality of the DNA extraction was assessed by 1% agarose gel electrophoresis, and DNA concentration and purity were determined using a spectrophotometer (NanoDrop 2000, Thermo Scientific). The primers ITS1F(5’-CTTGGTCATTTAGAGGAAGTAA-3’) and ITS2R(5’-CTTGGTCATTTAGAGGAAGTAA-3’) were used to amplify the ITS variable region of the fungal rRNA gene, and each sample was repeated three times. Finally, PCR products from the same sample were mixed, and high-throughput sequencing using an Illumina MiSeq 300 sequencing platform (San Diego, USA) (Megbio, Shanghai, China). Original disembarkation data were obtained by sequencing for filtering and quality assessment. After distinguishing the samples, non-repetitive sequences (excluding single sequences) were analyzed using operational taxonomic unit (OTU) clustering with 97% similarity. After removing the chimera during the clustering process, OTUs represented the DNA sequence. Based on the UNITE 8.0 database (http://unite.ut.ee/index.php), annotation information was obtained by comparing the representative sequences using the Ribosomal Database Project (RDP) classifier Bayesian algorithm. Following comparison, the sequence number of the soil fungal community was flattened according to the minimum sample size. The raw sequences of fungal high-throughput sequencing for this thesis have been uploaded and stored in the National Center for Biotechnology Information (NCBI) database, with the project number PRJNA1068063.

### 2.4 Statistical analysis

Before conducting statistical analysis, we used the Shapiro-Wilk test to check the normality of the data. For non-normally distributed data, a logarithmic transformation was applied to make it approach a normal distribution. Data analysis was used to conduct R software (version R 4.2.1; https://www.r-project.org/). Using Duncan’s test, we conducted a comparative analysis of the soil physicochemical properties under different vegetation types. Using the T-test, we investigated the differences in fungal community α-diversity (Shannon-Wiener diversity index, Chao index) among various vegetation types. *β* diversity of soil fungal communities in different vegetation restoration patterns was analyzed by a non-metric multidimensional scale (NMDS) based on the Bray-Curtis distance, and the significance of differences between groups under different vegetation restoration patterns was tested using Anosim analysis (Xiao et al., 2022). Pearson’s correlation analysis were used to analyze the correlation between soil factors and soil fungi communities (Chen et al., 2020). Additionally, distance-based redundancy analysis (db-RDA) was performed using Bray-Curtis distance to explore the relationship between species and soil physicochemical properties.

Co-occurrence network analysis was performed based on different soil samples (Yuan et al., 2021). The Spearman correlation coefficient that is higher than 0.1% OTU of the relative abundance of all samples was calculated using the psych package in R, and only values of *r* > 0.7 and *P* > 0.05 were retained for molecular network construction. Gephi 0.9.2 software (WebAtlas, Paris, FRA) was used to calculate the topological properties of network, such as network nodes, edges, average weight degree, average path length, average aggregation coefficient (Li et al., 2023). Null-model analysis was been used to determine the community-building mechanisms of fungal communities (Jiao and Lu, 2020). Before proceeding with community assembly, the “microeco” package is utilized to plot Mantel correlograms to test whether the phylogenetic signal of the fungal community is significant (Stegen et al., 2013). The relative importance of stochastic and deterministic processes was quantified in terms of β-nearest taxon index (β-NTI) and Raup-Crick (RCbray) (**Table 1**) (Ning et al., 2020).

**Table 1:** Assembly processes of microbial community based on null model.

Parameters		Assembly process
β-NTI	RCbray	
β-NTI < −2		Homogeneous selection
/β-NTI/< 2	RCbray <−0.95	Homogeneous dispersal
	/RCbray/< 0.95	Undominated process
	RCbray > 0.95	Dispersal limitation
β-NTI > 2		Heterogeneous selection

## 3 Results

### 3.1 Community structure of soil fungi under different vegetation restoration patterns

**Figure 2:**
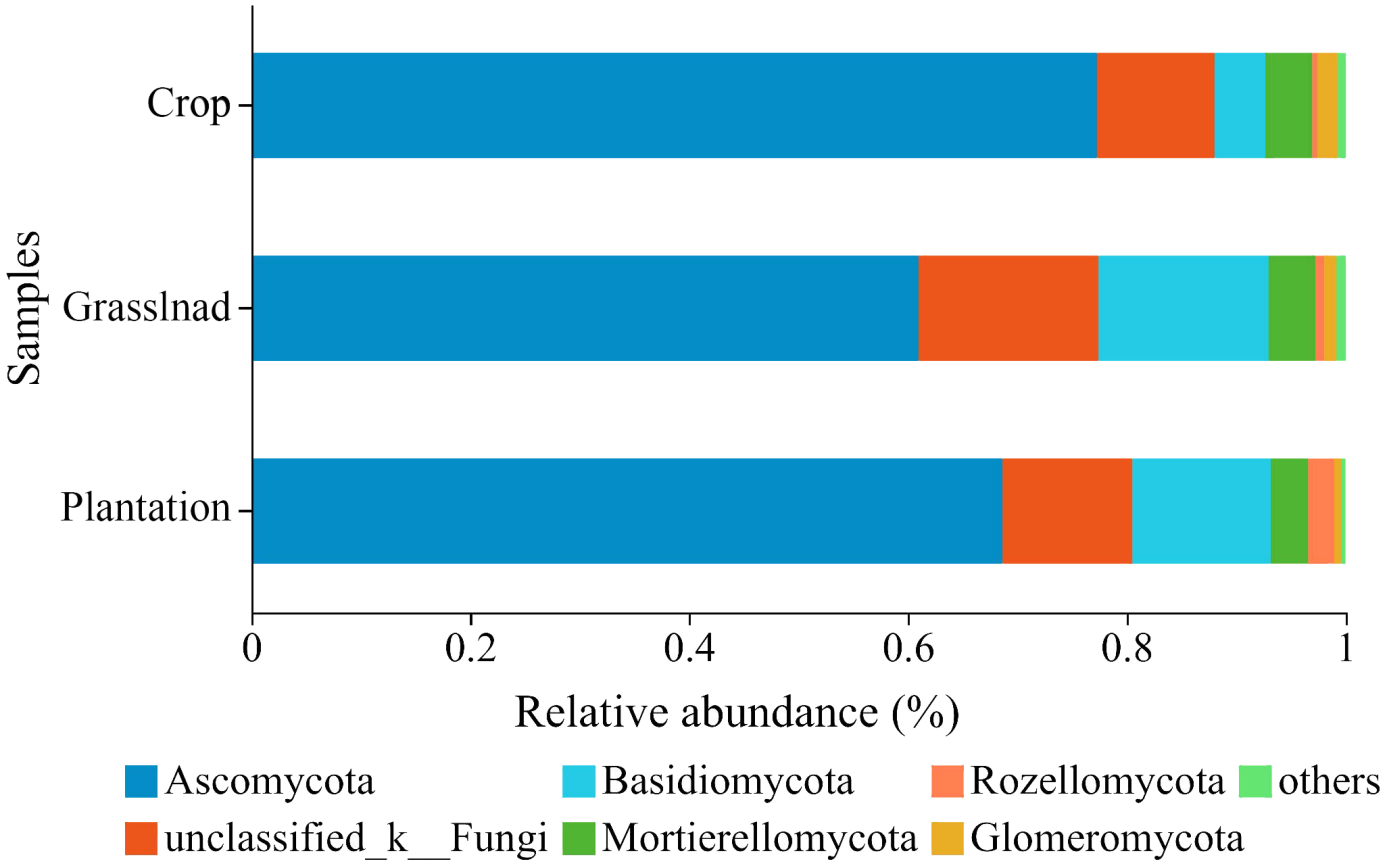
Relative abundances of soil fungal community at the phyla level in different vegetation restoration patterns.

The three vegetation restoration patterns shared 1,095 OTUs, with 871, 897, and 1125 OTUs belonging to crop, grassland, and plantation, respectively (**Supplementary Figure S1**). At the phylum level, the relative abundance of Ascomycota in the different vegetation restoration patterns exceeded 60%, of which 77.29%, 60.97%, and 68.64% were found in the crop, grassland, and plantation, respectively (**Figure 2**). The relative abundance of Basidiomycota in the crop was 4.61%, which was lower than that in the grassland (15.52%) and plantation (12.68%) (**Figure 2**). At the same time, there are also a considerable number of unclassified_k_fungi in all three vegetation restoration modes, with their respective relative abundances >10%.

**Figure 3:**
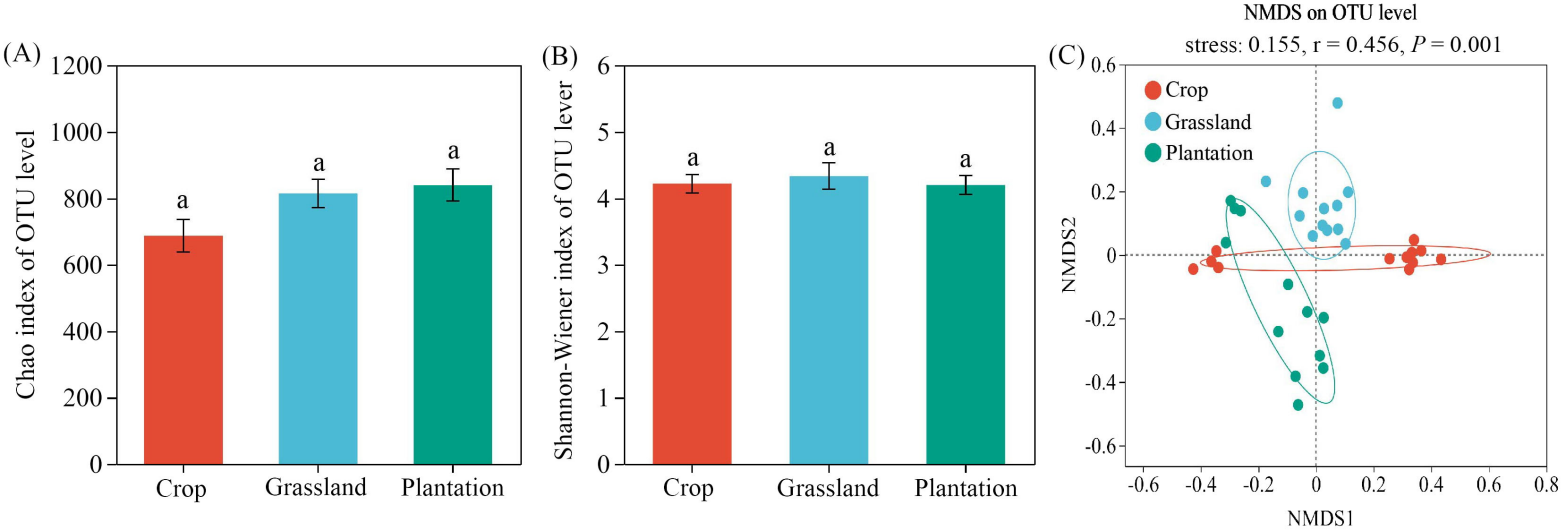
Diversity of Chao1 index **(A)**, Shannon-Wiener index **(B)** and Non-metric multidimensional scaling (NMDS) **(C)** of fungal communities in different vegetation restoration patterns. The significances were examined using the Anosim tests (*P*  <  0.001). Different letters in the figure indicate significant differences at the *P* < 0.05 levels.

The Shannon-Wiener index (4.20-4.33) and Chao index (687.20-839.70) of the fungal communities were not significantly different among the three vegetation restoration patterns (Kruskal-Wallis test, *P* > 0.05) (**Figures 3A, B**). NMDS indicated a significant separation of soil fungal communities among the three vegetation restoration patterns (r = 0.456, *P* = 0.001, stress = 0.155) (**Figure 3C**).

### 3.2 Relationship between soil fungal community and soil properties

**Figure 4:**
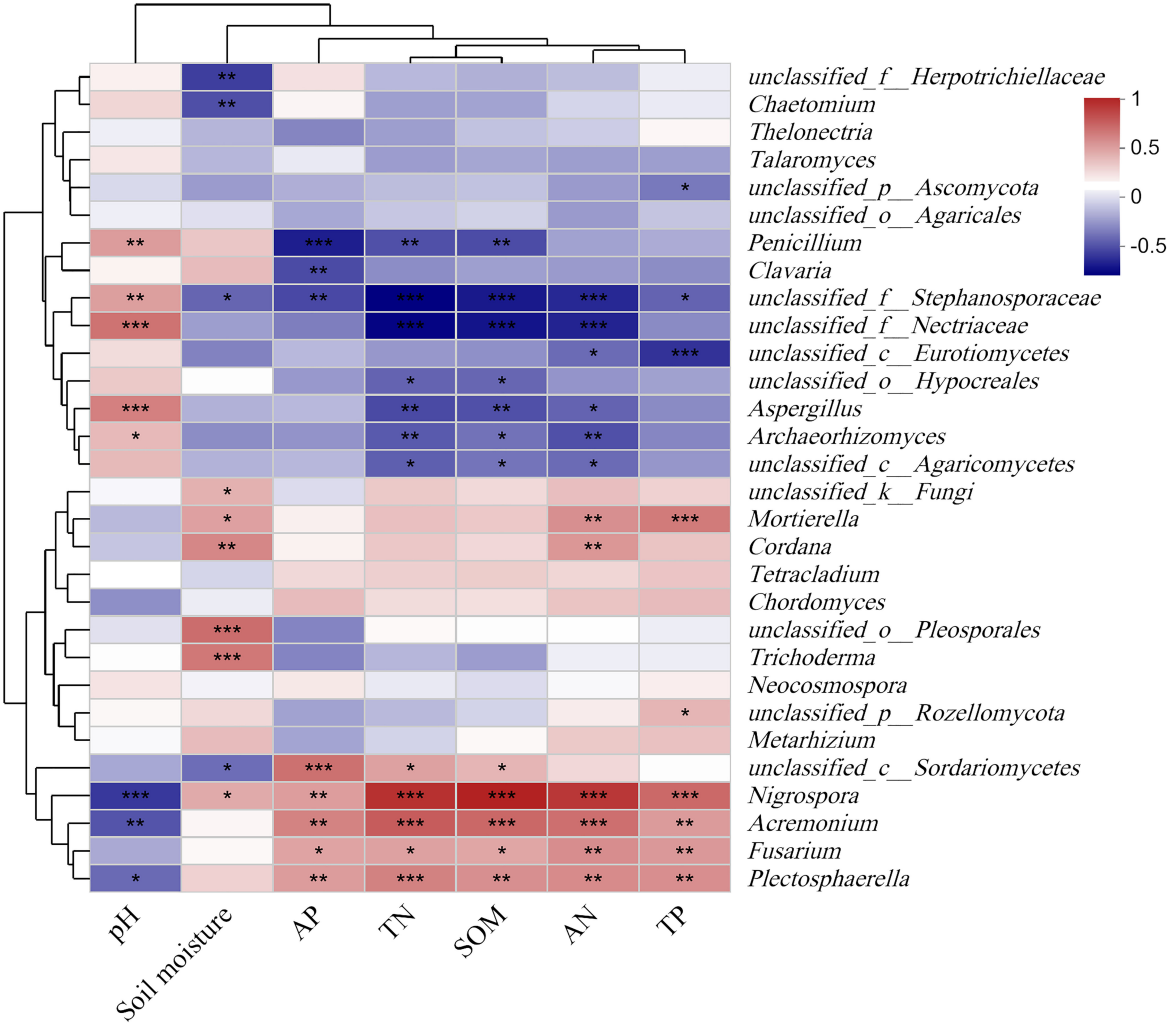
Relationships between the relative abundance of fungal communities and chemistry properties of soil. *** *P* < 0.001, ** *P* < 0.01, * *P* < 0.05.

The soil moisture of grassland and plantation increased significantly compared with that of crop. The soil TN, AN, TP, and SOM content in grassland soils were significantly higher than those in crop and plantation, while soil AP content in the crop was higher than that in grassland and plantation. At the same time, the soil pH in the grassland is the lowest (**Supplementary Table S1**). At the genus level, there was a significant correlation between some species in the top 30 most abundant soil fungi and soil properties (**Figure 4**). *Nigrosspora*, *Acremonium*, *Fusarium* and *Plectosphaerella* were significantly and positively correlated with SOM, AP, TN, AN, and TP in the soil, and significantly and negatively correlated with soil pH. Meanwhile, some fungal groups, such as *Neocosmospora*, *Chordomyccs*, *Tetracladium*, *Thelonectria*, *Talaromyces*, etc. showed no significant correlation with soil properties (**Figure 4**).

**Figure 5:**
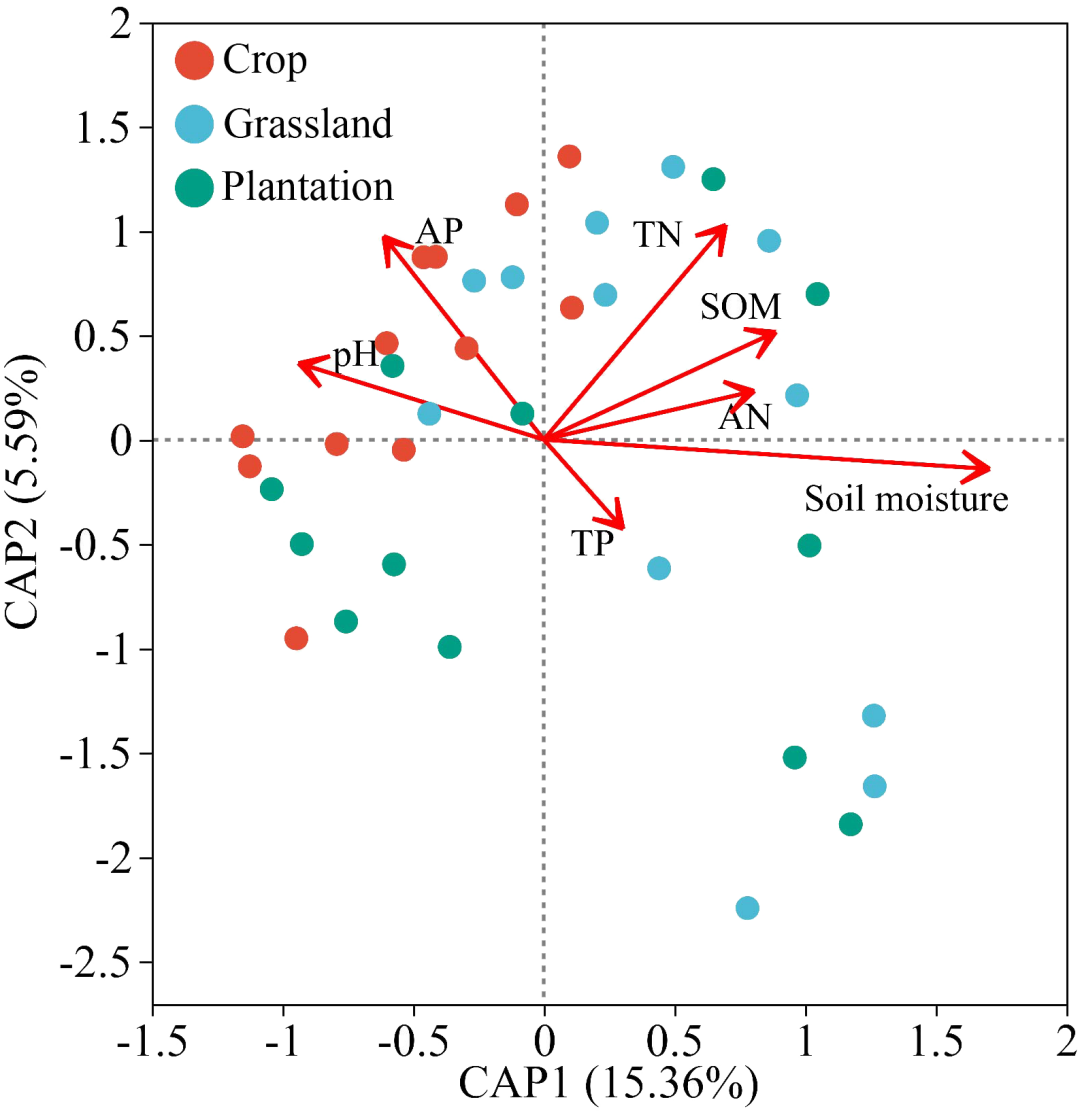
Relationships between chemistry properties of soil and composition of fungal communities using distance based on redundancy analysis (db-RDA) in different vegetation restoration patterns.

At the phylum level, the db-RDA analysis demonstrated that soil properties explained 20.45% of the relative abundance variation amongst the top 30 soil fungi. The first and second axes of the db-RDA explained 15.36% and 5.59% of the variation in relative abundance, respectively (**Figure 5**). Moreover, the relative abundance of fungi in the karst soil was positively correlated with soil moisture (*P* = 0.001, *R* ^2^ = 0.995) and negatively correlated with soil TN (*P* = 0.023, *R* ^2^ = 0.201).

### 3.3 Co-occurrence network of fungal communities under different vegetation restoration patterns

**Figure 6:**
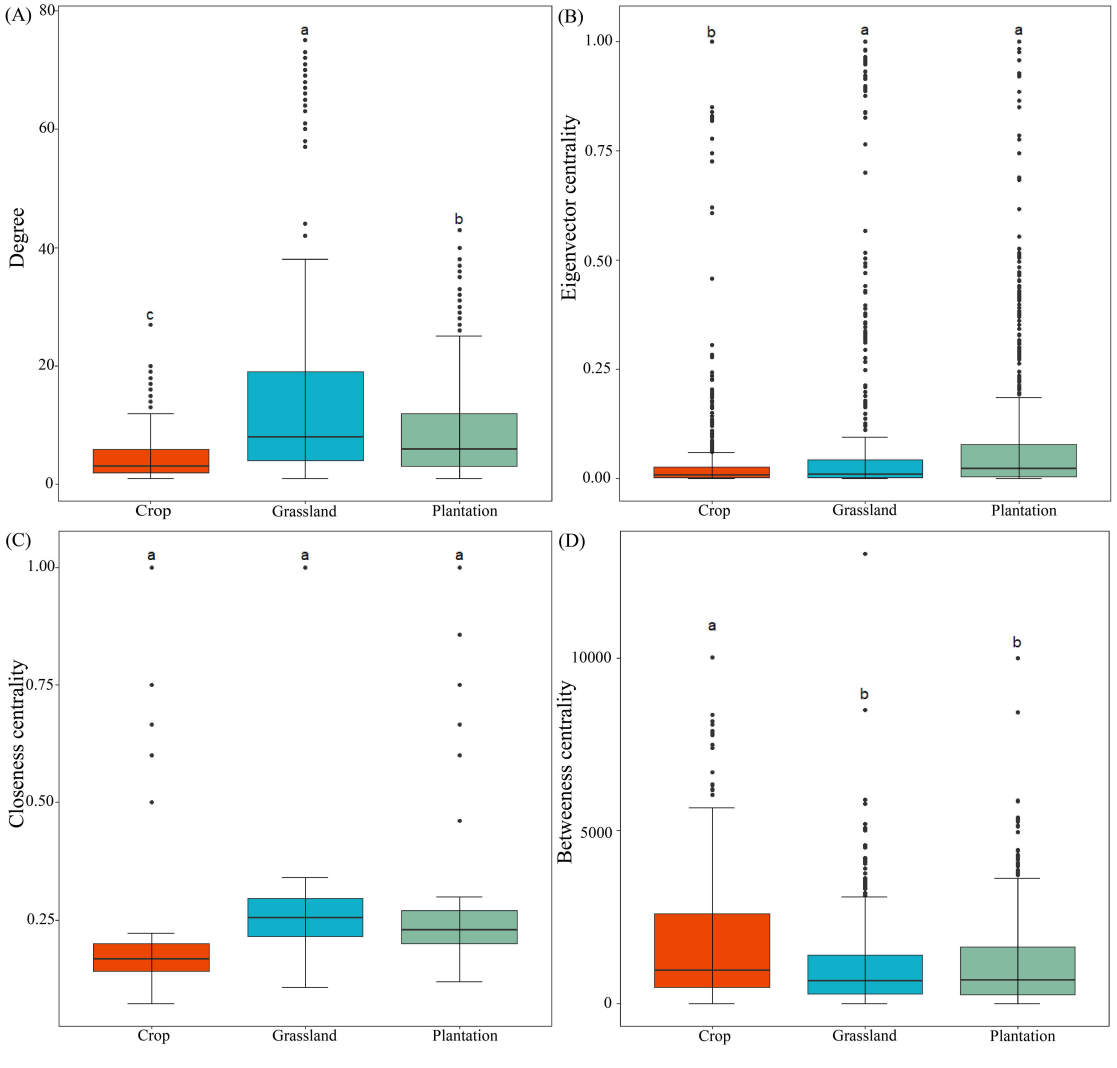
**(A–D)** Comparison of topological features in different vegetation restoration patterns. Different letters indicate significant differences at the *P* < 0.05 level.

**Figure 7:**
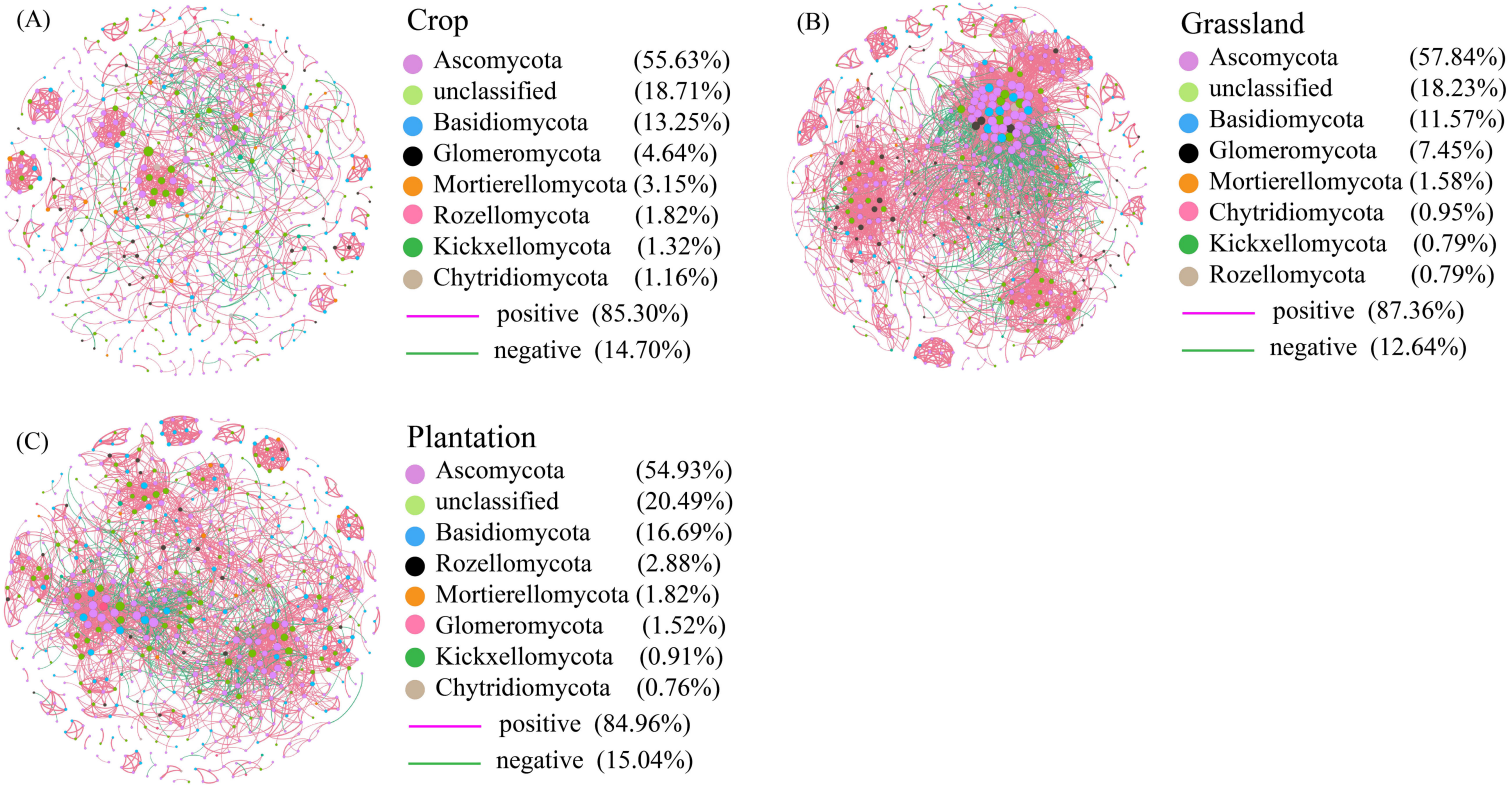
**(A–C)** The co-occurrence network of fungal taxa in different vegetation restoration patterns. The positive and negative correlations in different places are indicated by pink and green lines, respectively. Node size indicates the correlation between nodes. Circles with the same color nodes suggest different fungal OTUs that are the same phylum.

The co-occurrence network of all fungal communities in karst soil had a power-law *R* ^2^ value of 0.721-0.834, which fits into a typical scale-free network (**Supplementary Figure S2**). In all co-occurrence networks, most of the nodes were peripheral nodes, and no nodes belonged to the center of the network (**Supplementary Figure S3**). Compared with co-occurrence networks in crop, the nodes, edges, and average weight degrees were higher in grassland and plantation (**Table 2**), indicating that the co-occurrence networks in grassland and plantation had a larger scale and more complex interactions. Compared with co-occurrence networks in grassland and plantation, crop co-occurrence network has longer average path lengths (**Table 2**), which implied that it has looser network structure and is less efficient in transferring material, energy, and information between species. In addition, the degree of the co-occurrence network in grassland were highest, and the betweenness centrality in the crop was the highest. There were no significant differences in the closeness centrality of the co-occurrence network in all the vegetation soils (**Figure 6**). The connections in all fungal network diagrams were mainly positively correlated, indicating that the fungal communities in the soil were primarily symbiotic under different vegetation restoration patterns. Interaction of the fungal community was the strongest in grassland (87.36%) and lowest in plantation (84.96%). In addition, the dominant OTU in the soil in the three restoration patterns belonged to Ascomycota (**Figure 7**).

**Table 2:** Co-occurrence network properties within soil fungal taxa in different vegetation restoration patterns.

Land types	Crop	Grassland	Plantation
Node	604	631	659
Edge	1340	4801	2872
Average weighted degree	3.955	13.635	7.730
Modularity	0.830	0.566	0.719
Average clustering coefficient	0.463	0.420	0.555
Average path length	6.779	4.326	4.732
Density	0.007	0.002	0.013

### 3.4 Assembly mechanism of fungal communities under different vegetation restoration patterns

**Figure 8:**
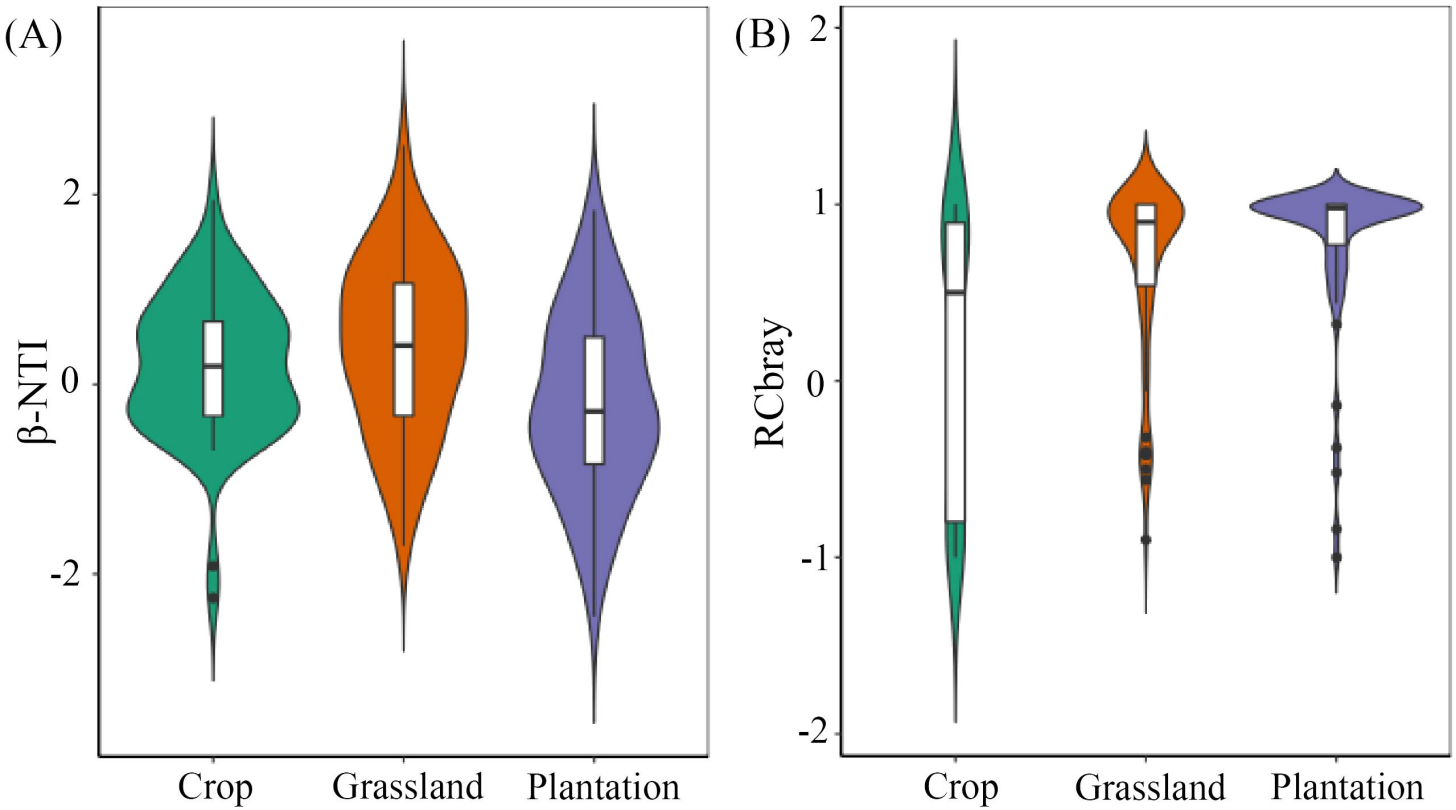
The difference index of β-NTI **(A)** and RCbray **(B)** based on null model in different vegetation restoration patterns. The bottom and top of the boxes the represent 25% and 75%, respectively. The white circles and central black lines represent medians and means, respectively.

Phylogenetic Mantel correlogram revealed a significant positive phylogenetic signal across short phylogenetic distances (**Supplementary Figure S4**). All soil fungal communities with β-NTI < 2 (**Figure 8**), implying that randomness was the main factor affecting the assembly of all fungal communities. In soil fungal communities of crop,/RCbray/≤ 0.95 indicated a undominated process. However, RCbray ≥ 0.95 in grassland and plantation demonstrated that dispersal limitation was a community assembly process of soil fungi.

## 4 Discussion

### 4.1 Diversity and structure of fungal communities

Here, we found that there was no significant change in the α diversity of soil fungal communities under different vegetation restoration patterns (**Figure 2**), which may indicate that some fungi had mycelial networks (de Vries et al., 2012; Barnard et al., 2013) or a specific mode of reproduction (spore reproduction) (Roper et al., 2010; Adams et al., 2013; Li et al., 2020; Wang et al., 2020). The robust adaptability of the fungal community, characterized by a relative insensitivity to environmental changes, may be a contributing factor to the observed stability in α-diversity (Barnard et al., 2013). In addition, the *β* diversity of fungal communities in karst soil differed significantly between different vegetation restoration patterns, which is consistent with previous research (Zhang et al., 2022c). The alteration of litter, root exudates, and soil characteristics may synergistically affect the fungal community structure (Prescott and Grayston, 2013; Ding et al., 2022).

Ascomycota and Basidiomycota were the most abundant fungal phyla in soil, which is consistent with the results of other studies (Dang et al., 2017; Liu et al., 2019; Chen et al., 2020). The relative abundance of Ascomycetes decreased during the conversion of crop to grassland and plantation, which is also consistent with the previous results (Dang et al., 2017; Wang et al., 2022). This may be because Ascomycetes have strong stress resistance and competitiveness and can better utilize soil nutrients in fields with strong disturbance (Ventorino et al., 2016; Egidi et al., 2019). Moreover, the relative abundance of Basidiomycota increased significantly after returning crop to plantation and grassland, which is similar to previous studies (Dang et al., 2017). This change may be because Basidiomycota produces carbohydrate-active enzymes (CAZymes) that promote litter decomposition into the soil, which increases the litter content after returning crop to plantation and grassland and promotes nutrient absorption and utilization by the mycorrhiza and plant root community (Baldrian, 2017; Zhang et al., 2018, 2022). In addition, the proportion of unclassified_k_Fungi in the three vegetation patterns ranged from 10.78% to 16.48%, The identification of unclassified_k_Fungi requires improvement of the soil fungal taxa library in karst areas.

### 4.2 Relationship between soil fungal communities and soil factors under different vegetation restoration patterns

Previous studies indicated that drought treatment significantly affects the community composition of total fungi, as well as the abundance and rarity of fungi (Xu et al., 2022). Fungal groups in soil (at the genus level) were significantly correlated with soil physicochemical factors (**Figure 5**), which is consistent with the previous results (Mendes et al., 2015; Tian et al., 2017). Soil moisture affects oxygen content and gas diffusion in soil, which may result in differences in the growth of fungal taxa under different vegetation cover patterns (Freeman et al., 2002). In addition, soil pH and SOM were considered the main factors shaping soil fungal communities (Zeng et al., 2020). However, some research indicated that neither pH nor TC content significantly affects the fungal community structure (Chen et al., 2020), aligning with the findings of this study.

TN was the dominant factor influencing the structure and distribution of soil fungal communities in the karst region (**Figure 6**), consistent with findings in the Loess Plateau and desert regions of China (Li et al., 2019; Xu et al., 2022). TN is the predominant factor influencing the structure and distribution of soil fungal communities in the karst region (**Figure 6**), consistent with research findings from the Loess Plateau and desert areas. This is likely due to nitrogen (N) facilitating synergistic interactions between fungi and plant roots through fungal hyphae, thereby enhancing plant efficiency in utilizing nitrogen in the soil (Veresoglou et al., 2012; Kumar and Atri, 2018). Some studies suggest that fertilization may increase the proportion of nitrogen cycling microorganisms, exhibiting higher nitrogen turnover activity in response to long-term increases in nitrogen fertilizer intensity (Wang et al., 2018). Although some studies indicated that soil phosphorus (P) and potassium (K) significantly affected fungal composition and distribution (Li et al., 2019; Ji et al., 2020), however, the fertilization measures in this study did not significantly impact the fungal community. Additionally, the influence of soil nutrients on fungal communities was also subject to interactions with plant factors (such as species and root exudates) and soil factors (such as temperature and moisture) (Jiao et al., 2018; Jiao and Lu, 2020; Xiao et al., 2022; Xu et al., 2022).

Our data indicate that the explanation rate of soil to fungal communities was lower than that in similar studies (Xu et al., 2021), which may be because this study only discussed the influence of soil properties on the fungal community. Fungal communities may be influenced by factors such as root exudates, litter, and soil depth (Zhu et al., 2012; Prescott and Grayston, 2013). To better understand the fungal community in karst-degraded heterogeneous habitats, the above factors should be further considered in future studies.

### 4.3 Vegetation restoration affected the interaction of soil fungi species

Stable ecosystems have complex networks that maintain the structure and function of the soil ecosystem (Toju et al., 2018; Yuan et al., 2021; Ding et al., 2022). Analysis of microbial co-occurrence network and their influencing factors is key to understanding the response of microbial ecosystems when returning crop to plantation and grassland (Qiu et al., 2021; Li et al., 2023). We found that the number of network connections of fungal communities in crop was the lowest, and the microbial interactions in the soil were weakened, which may be due to the low modularity value lessens niche differentiation (Faust and Raes, 2012). The scale of soil fungal networks in grassland and plantation are larger than that of crop, which may reinforce effectively microbial flora to utilize soil nutrients (Ma et al., 2020). At the same time, it has been found that complex networks with higher connectivity and functional redundancy, and can promote the ecosystem services provided by fungal communities (Mougi and Kondoh, 2012; Sheng et al., 2017). Previous research has indicated that positive microbial interactions may increase the connections within microbial communities and their responses to pressure, and these positive interactions, in turn, provide buffers against environmental disturbances (Coyte et al., 2015; Zhao et al., 2022). This study showed that positive interactions of soil fungal communities in grassland were the highest (87.36%), leading to the highest resources sharing (Milici et al., 2016). This may be related to niche sharing and enhanced microbial interactions caused by higher grass cover density (Qiu et al., 2022).

In microbial co-occurrence networks, key microbial taxa represent highly interconnected communities, described as connectors, which indicates environmental changes (Banerjee et al., 2018). In the three restoration patterns, Ascomycota appeared as the key fungal group in the networks and had the highest relative abundance in the community, indicating that Ascomycota had a strong connection with other associated taxa in the microbial network, influencing the community composition through its impact on other related groups (Olesen et al., 2007).

### 4.4 Assembly process of soil fungal communities under different vegetation restoration patterns

The assembly of communities is governed by both deterministic and stochastic processes, but their relative importance varies across different environments (Tian et al., 2017). Our results showed a/β-NTI/of < 2 in all soil fungal communities, suggesting that stochasticity dominates the assembly of fungal communities, which is consistent with the mechanism of microbial community construction in lakes, reservoirs (Liu et al., 2015), and forests (Zou et al., 2023). Meanwhile, the/RCbray/of soil fungal communities in grassland and plantation was < 0.95, meaning that the assembly process was a dispersal limitation, whereas that in crops was > 0.95, indicating that the construction process was an undominated process Compared with the biomass of grass and plantations, the biomass of crop and restriction of soil nutrients are relatively small (Xiao et al., 2019), which may explain why undominated processes play a dominant role in the construction of fungal communities in crop. The strong spatial heterogeneity of soil nutrients and moisture in grassland and plantation, caused by developed roots (Chen et al., 2020), maybe the reason why dispersal limitation is dominant in the community assembly of soil fungi.

Stochastic processes are more important at the local scale with less environmental change, and deterministic processes are generally thought to dominate the assembly of large-scale microbial communities (Wang et al., 2013; Shi et al., 2018). Although this study found that the vegetation restoration model affected the community assembly process of karst soil fungi at the plot scale, further research on the effects of temperature and precipitation on soil microbial community construction at regional and global scales is needed. In addition, the nature of soil organisms (category, size) interacts with the ecological processes of community assembly, which renders the mechanism of karst vegetation restoration on karst soil ecosystem restoration unclear. Therefore, it is necessary to integrate vegetation, soil microorganisms, soil animals, and their interactions in future research on the soil biodiversity and restoration mechanisms of karst ecosystems.

## 5 Conclusions

Implementation of the Grain to Green Project, the *β* diversity of the soil fungal community was altered in karst region. The relative abundance of Ascomycetes had decreased, while the relative abundance of Basidiomycota increased, and a large number of unclassified_f_Fungi existed in karst areas. Soil moisture and TN were the main environmental factors affecting the relative abundance of fungal communities. From crop to grassland and plantation, the assembly mechanism of the fungal community changed from an undominated to a dispersal limitation. With the restoration of karst vegetation, the scale and complexity of the co-occurrence network of fungal communities increased. Our data indicate that the restoration of karst vegetation has a significant impact on the diversity, co-occurrence network, and community assembly processes of soil fungi, contributing to a deeper understanding of the mechanisms behind karst soil ecosystem restoration.

## Data Availability

The datasets presented in this study can be found in online repositories. The names of the repository/repositories and accession number(s) can be found below: https://www.ncbi.nlm.nih.gov/, PRJNA1068063.
