# Supplementary material for: Effect of different vegetation restoration patterns on community structure and co-occurrence networks of soil fungi in the karst region
Source: Front Plant Sci. 2024 Sep 4;15:1440951. doi: 10.3389/fpls.2024.1440951 (PMC11408217; doi:10.3389/fpls.2024.1440951)
Supplement: Supplementary file 1 [file DataSheet1.docx]

Supplementary Material


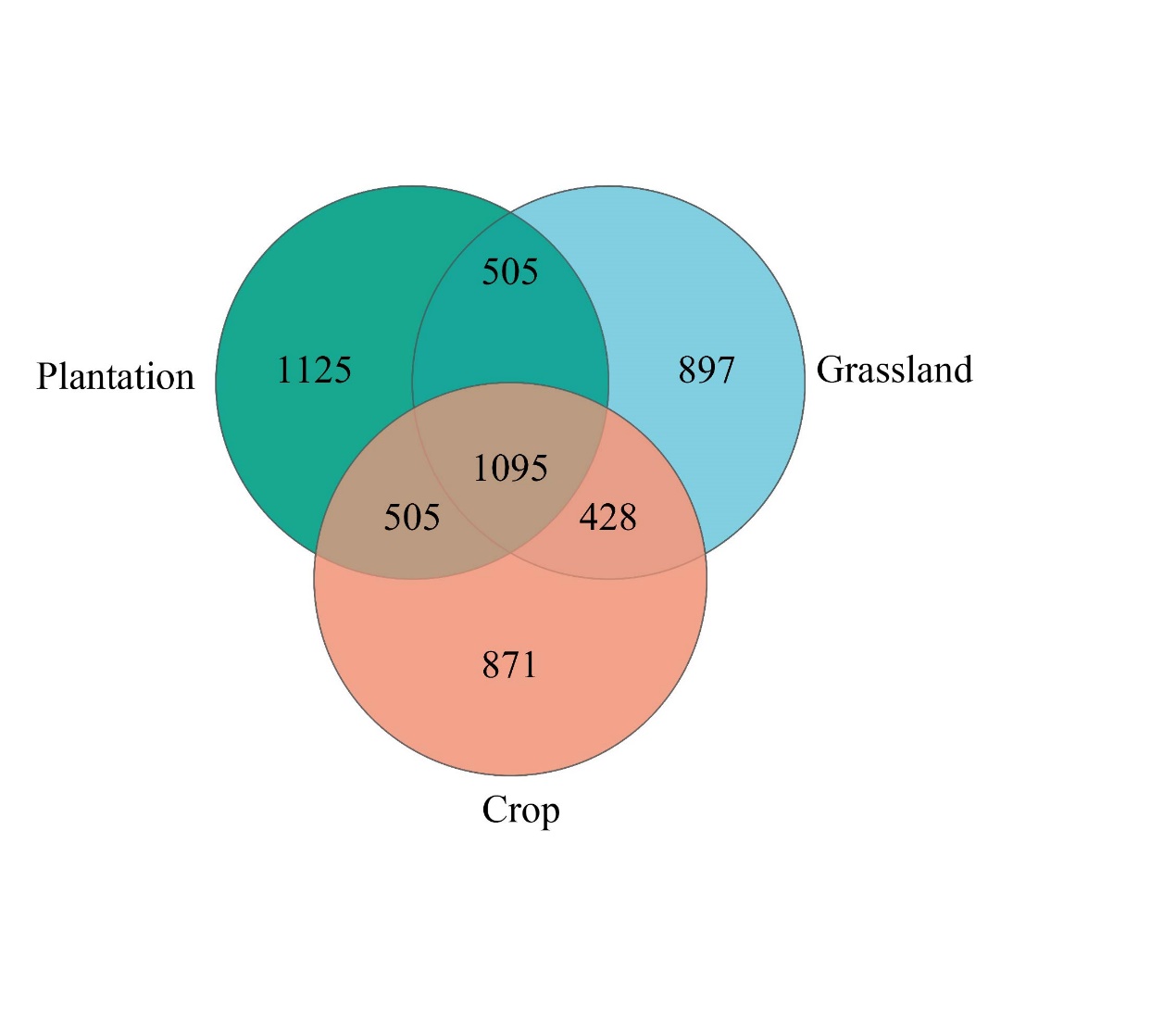


**Fig.** **S1**: Venn diagram illustrating the number of soil fungal OTUs in different vegetation restoration patterns.


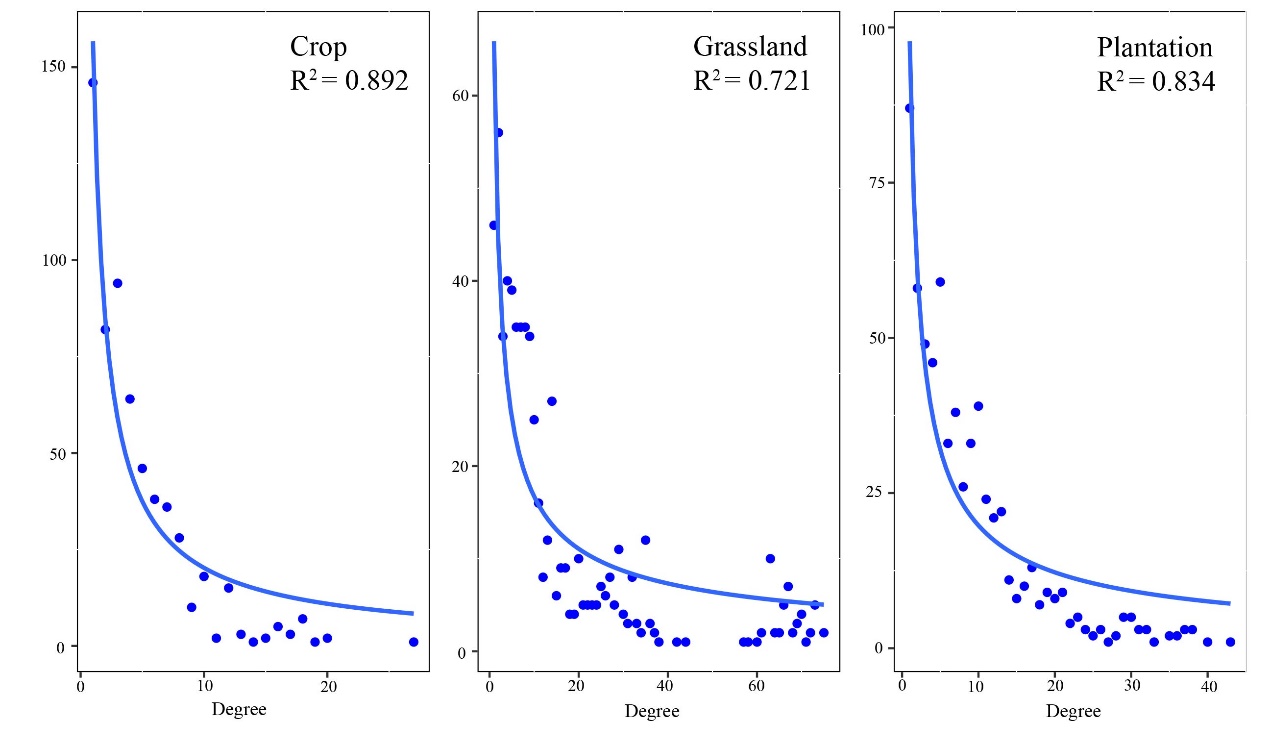


**Fig.** **S2**: Power law of fungal co-occurrence networks in different vegetation restoration patterns, R^2^ representing the goodness of fit of distribution.


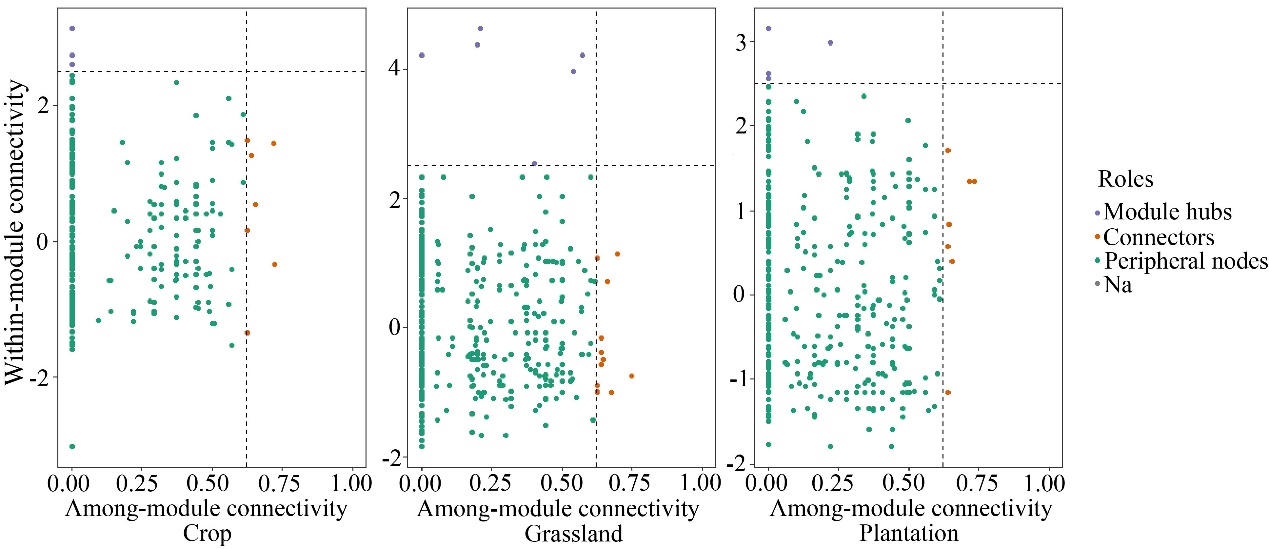


**Fig.** **S3**: Modules distribution of nodes based on topological roles in different vegetation restoration patterns.


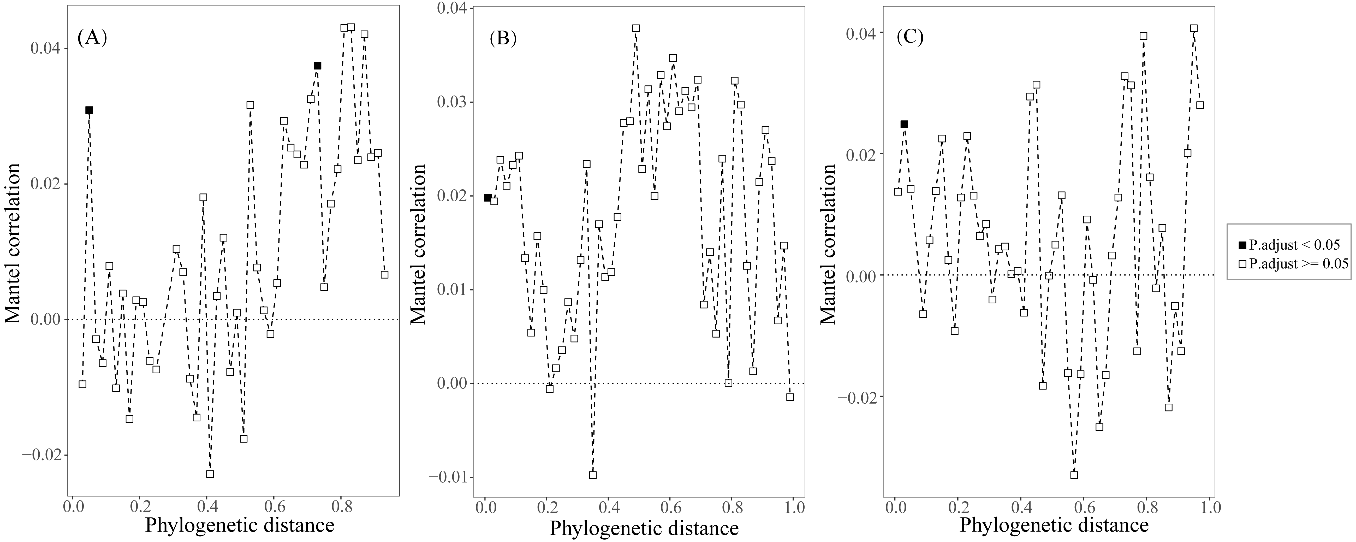


Fig S4 Phylogenetic Mantel correlogram for: top layer in crop (A), grassland (B) and plantation (C). The plots show significant phylogenetic signal across relatively short phylogenetic distances. Black squares indicate significant correlations (*P* < 0.05) after Bonferroni correction for multiple comparisons

**Table S1**: Properties of soil at 0–20 cm depth in different vegetation restoration patterns.

| Soil properties | Crop | Grassland | Plantation |
| --- | --- | --- | --- |
| pH | 7.92 ± 0.06a | 7.82 ± 0.14b | 7.99 ± 0.07a |
| TN (g/kg) | 4.32 ± 0.34b | 5.29 ± 0.44a | 2.78 ± 0.56c |
| AN (g/kg) | 0.33 ± 0.05b | 0.39 ± 0.02a | 0.31 ± 0.04b |
| AP (mg/kg) | 11.31 ± 2.72a | 7.62 ± 1.76b | 2.25 ± 0.51c |
| TP (g/kg) | 1.14 ± 0.09a | 1.26 ± 0.17a | 0.96 ± 0.25b |
| SOM (g/kg) | 77.40 ± 2.19b | 95.34 ± 8.97a | 76.57 ± 18.13c |
| Soil Moisture (%) | 21.84 ± 4.65b | 30.36 ± 4.76a | 27.20 ± 7.53a |

Different letters in the same row indicate that parameters show significant differences at *P* < 0.05 level by ANOVA. Data are mean ± standard deviation (n=12).
